# Using Crowdsourcing to Develop a Peer-Led Intervention for Safer Dating App Use: Pilot Study

**DOI:** 10.2196/12098

**Published:** 2020-04-21

**Authors:** William Chi Wai Wong, Lin Song, Christopher See, Stephanie Tze Hei Lau, Wai Han Sun, Kitty Wai Ying Choi, Joseph Tucker

**Affiliations:** 1 Department of Family Medicine and Primary Care Li Ka Shing Faculty of Medicine University of Hong Kong Hong Kong; 2 School of Biomedical Sciences Li Ka Shing Faculty of Medicine University of Hong Kong Hong Kong; 3 Division of Infectious Disease Department of Medicine University of North Carolina at Chapel Hill Chapel Hill, NC United States

**Keywords:** dating apps, intervention mapping, crowdsourcing, peer-led approach, sexual health

## Abstract

**Background:**

Smartphone-based dating apps are rapidly transforming how people seek potential sexual and romantic partners. However, they can also increase the risk of unsafe sexual behaviors, harassment, and infringement of personal privacy. Current research on interventions for safer dating app use remains insufficient.

**Objective:**

The goal of this study was to describe the development of an intervention for safer dating app usage using crowdsourcing and peer-led approaches.

**Methods:**

This paper describes the development of an intervention program designed to promote safer dating app use among college students. Crowdsourcing and peer-led approaches were adopted during key stages of the development process. Focus group discussions were held to assess the experience and needs of dating app users. A crowdsourcing contest then solicited ideas for performance objectives for the intervention. These objectives were grouped to further identify practical strategies. A one-day intensive workshop was subsequently held with peer mentors to brainstorm ideas for the production of creative interventional materials. The intervention programs were produced and tested in a pilot study. The app’s effectiveness will be evaluated in a cluster randomized controlled trial.

**Results:**

The intervention program consists of a risk assessment tool, a first-person scenario game, and four short videos. The risk assessment tool, comprised of 14 questions, will give the participant a score to determine their level of risk of adverse events when using dating apps. The scenario game is a first-person simulation game where the players are presented with choices when faced with different scenarios. The short videos each last 2-4 minutes, with points of discussion aimed at addressing the risks of using dating apps. The programs were piloted and were found to be relatable and helpful when further modifications were made.

**Conclusions:**

Potential challenges identified during the development process included data management and analysis, sustaining peer mentors’ interests and participation, and balancing between providing more information and perpetuating social stigma around dating app use. By integrating new approaches, such as crowdsourcing and the peer-led approach, in developing an intervention for safer dating app use, our development process provides a viable model for developing future interventions to address the risks associated with dating app use.

## Introduction

### Background and Motivation

The popularization of smartphone-based dating apps has drastically changed dating patterns and behaviors among young adults across the globe. Researchers from various countries have reported high percentages of dating app use among young adults of different genders and sexual orientations. In the United States, for instance, approximately 40% of sampled young heterosexual men and women used dating apps in 2017 [1], and the percentage for men who have sex with men is significantly higher at around 69% [2]. In Hong Kong, a 2016 study found an equally high proportion (52.9%) of dating app users among both heterosexual and homosexual college students [3].

By facilitating convenient interpersonal exchanges, dating apps broaden one’s personal and romantic networks and bring new freedoms, opportunities, and pleasures [4]. However, they also engender a range of potential risks in personal safety, privacy, and sexual health. A high correlation between dating app use and multiple sexual partners has been identified among lesbian, gay, transgender, and bisexual populations, which is an alarming indicator for sexual health–related risks, such as HIV or hepatitis C infection [5]. Some studies have also raised concerns about the lack of awareness around the protection of personal information on dating apps [6,7], highlighting potential privacy and safety risks.

Currently, resources are insufficient for dating app users in Hong Kong or elsewhere to understand and manage these risks. A review of a large number of smartphone-based dating apps found that the majority contained no information about sexual health or sexual assault, so users had low exposure to sexual health and personal safety content [8]. To address these issues, our research team engaged dating app users in Hong Kong to develop intervention programs for promoting safer dating app usage among college students. We implemented innovative approaches, including crowdsourcing and the peer-led approach, in various stages of the development of the intervention.

### Study Aims

This study offers a close, step-by-step investigation of using crowdsourcing and peer-led approaches to develop an intervention for safer dating app use. It aims to propose a viable model for developing similar dating app use interventions in the future. Potential challenges in the intervention development have been identified, and the effectiveness of adopting hybridized approaches was explored. This study also reports on the findings of a pilot study for the intervention.

## Methods

### Needs Assessment

The needs assessment is a systematic study of the discrepancy between what is and what should be in a group and situation of interest [9]. Data for the assessment can be collected through various channels, including a literature review, brainstorming, and focus groups conducted with the target population. Findings from the needs assessment are used to specify intended changes in individual behaviors and environmental conditions.

Since the popularity of dating apps is a relatively new phenomenon, literature on its risks remains limited. We, therefore, decided to conduct focus group discussions and engage app users in discussing their safety strategies to elicit insights into their behaviors, motivations, and perceptions when using dating apps. We recruited participants through a Hong Kong–based organization, “StickyRiceLove,” which specializes in peer-led, online, sexual health education. Inclusion criteria for participants included: experience with using a dating app, being between the ages of 18-35 years old, and able to speak Cantonese.

Each focus group was purposively constructed to include people of different genders, ages, and sexual orientations to facilitate heterogeneity in each focus group discussion and provide potential contrasts in discussions [10]. Focus group discussions of 6-8 people were conducted until data saturation was achieved, resulting in four groups (N=25 participants) in total. More demographic information can be found in Table 1.

**Table 1:** Needs assessment participants’ demographics (N=25).

Demographics		n
**Gender**		
	Male	15
	Female	10
**Age group (years)**		
	18-20	4
	21-23	4
	24-26	7
	27-29	4
	Over 30	6
**Sexual Orientation**		
	Heterosexual	17
	Homosexual	6
	Queer	1
	Bisexual	1
**App use experience (years)**		
	0-1	7
	2-3	8
	4+	10
**Instances of meeting friends via the app, n**		
	0-1	3
	2-5	7
	6-10	5
	>10	9

We used a semistructured interview guide developed from the literature to conduct the discussions (see Multimedia Appendix 1). Open-ended questions on dating app user experience, motivation, beliefs, and behaviors regarding intimacy and risk were used to facilitate discussion. Audio recordings were transcribed and translated from Cantonese to English.

These data were analyzed using thematic analysis, commencing with an open-coding approach of repeated line-by-line reading of the text and condensing of concepts, events, and meanings into representative codes. Two members of the research team (CS, STHL) independently undertook the coding to increase the robustness. Discrepancies between the initial coding were discussed and clarified with input from other members of the research team. Upon further cycles of analysis, themes were elicited from the data by constantly comparing the interview transcripts (see Multimedia Appendix 2).

Notably, users reported uncertainty and worry about potentially risky sexual health behaviors, which spanned from casual sex, multiple partners, or open relationships. They also expressed concerns about data privacy, including fear of blackmail using intimate data from dates or private photo exchanges from apps. Furthermore, we found that dating app users experienced several financial scams, some of which resulted in monetary loss. These findings posed key questions to consider for the intervention: How do we help dating app users in improving their sexual health knowledge, skills, and behaviors? How can we encourage dating app users to practice data safety and privacy while building relationships? How do we alert dating app users to the risks of financial scams and empower them by providing practical strategies? Apart from these questions, the participants also suggested that the experience of stigma and stereotyping remains a problem in the local dating app user community and that the intervention should provide reliable information and promote behavioral skills while avoiding further stigmatization.

Based on the findings from the focus group, we determined four prominent domains for intervention: sexual health, the security of personal privacy, sexual harassment and assault, and monetary scams. These domains are generally in line with existing literature on dating apps, although some of them are more specific to the Hong Kong context. The information gathered from the focus groups substantially contributed to the needs assessment. The research team then reviewed relevant theoretical and empirical literature and produced five behavioral outcome goals, as outlined in Table 2. We then further identified the determinants for each goal to pave the way for a more systematic approach in developing practical strategies in the next step.

**Table 2:** Behavioral outcomes and determinants.

Behavioral Outcomes	Examples (Determinants)
Users will understand the general benefits and risks associated with dating apps.	Name benefits and risks of dating apps (Awareness).
Users will evaluate relationship development and follow safe dating practices. Users will know the importance of safe sex and use condoms correctly and consistently when having sex.	Recognize risks of dating with people met on apps (Risk Perception). Select safe dating venues (Skills). Confidence in communicating personal limits and intentions on dates (Self-efficacy). Be aware of sexual health risks (Risk perception). Acknowledge condom use as effective protection against STIs^a^ (Attitude). Negotiate using condoms (Skills). Use condoms correctly (Skills). Confidence in using condoms (Self-efficacy). Confidence in choosing not to have sex without condom use (Self-efficacy).
Users will use practical skills to protect personal information and privacy.	Understand what should be categorized as sensitive personal information (Awareness). Acknowledge personal privacy risks in dating app use (Risk perception).
Users will be more aware of and better protect themselves against financial scams on dating apps.	Identify and avoid potential financial scams (Skills).

^a^STI: sexually transmitted infection.

### Crowdsourcing Contest

The next task was to select evidence-based methods to translate them into practical strategies effective for the target population. We combined crowdsourcing with the peer-led approach to tap into the collective knowledge of dating app users by hosting a crowdsourcing contest. The “‘Hi, Stranger!’ Dating Apps Education Design Contest” was held between November 2017 and February 2018, and dating app users were encouraged to submit online multimedia content (eg, texts, images, and videos) that addressed the risks and coping strategies used in dating app use. The contest was promoted through three channels: social network outlets such as Facebook and Instagram, local nongovernmental organizations focusing on sex education, and a large general education course on sexuality and culture at a local university, where we invited students to submit a website about dating app use in Hong Kong as part of their final projects. Our recruitment of participants ensured that while they were from diverse ethnic, cultural, educational, and class backgrounds, they were of similar age to the target population and had experience with dating apps.

We received a total of 24 group submissions in the form of websites from 127 participants. The websites covered a wide range of topics, from relationship-building to sexual harassment and dating violence. Apart from reviewing media reports and online resources, participants also drew on surveys and in-depth interviews that they conducted with their peers. More data about the crowdsourcing process can be found in Textboxes 1 and 2.

**Demographics of the crowd for the crowdsourcing contests.**

**Size of crowd**

- 127 participants (24 group submissions)

**Age**

- 17-22 years old

**Gender**

- Unspecified

**Education**

- College-educated

**Racial/ethnic background**

- Mixed, but mostly Asian.

**Relationship to the research question**

- Dating app users themselves.

**Referral source**

- Social media and classroom promotion of the crowdsourcing contest

**Logistics of the crowdsourcing.**

**Length of time**

- Four months

**Complexity of the task**

- Participants were asked to create multimedia and Web-based materials related to dating app use.

**Web platform**

- We recommended websites be created with the free Web-based platform wix.com.
- Submissions were made electronically in the form of website URLs.

**Incentives offered**

- A prize of HK $2000 (US $255) was offered for the best submission

**Data collected**

- A total of 24 submissions were received in the form of websites.

### Peer-Led Intervention Development

Upon collecting data from the crowdsourcing contest, we held a peer-led workshop to select practical strategies and produce intervention materials. We cooperated with StickyRiceLove to recruit peer educators from their pool of volunteers. A total of 12 peer educators were recruited who were between the ages of 20-30 years old and who had extensive experience in working on sex education projects. A one-day intensive workshop was held to select practical strategies and prepare for the production of intervention materials.

We briefed the peer educators about perceived benefits and risks associated with dating app use in Hong Kong, the purpose of the intervention, and the performance objectives. The peer educators were then divided into three groups to look into the websites from the crowdsourcing contest. They were given sufficient time to examine and discuss the different elements on the website both within and between groups. Through this process, the peer educators were able to identify the most effective and practical strategies based on the outcome goals, which included a risk assessment tool, a Web-based first-person simulation game that communicates benefits and risks of dating app use and coping strategies, and four short videos promoting risk perception, awareness of and skills for personal safety protection, and self-efficacy.

After the components of the intervention were determined, we adopted the Peer-Vetted Creative Production (PVCP) approach in producing intervention materials. Theorized in Brabham’s four-type typology for crowdsourcing, the PVCP approach is recommended for combining crowdsourcing and peer-led approaches to create and select creative ideas. It is ideal for ideation problems where solutions are highly sensitive to the taste of the target group [11]. We allocated time for peer educators to conduct brainstorming sessions and group discussions, through which they produced the format and scenarios for the game and themes and frameworks for the videos. These frameworks were taken up by the research team, who collaborated with sexual health professionals, volunteer actors and actresses, and photographers to produce the intervention materials.

**Figure 1:**
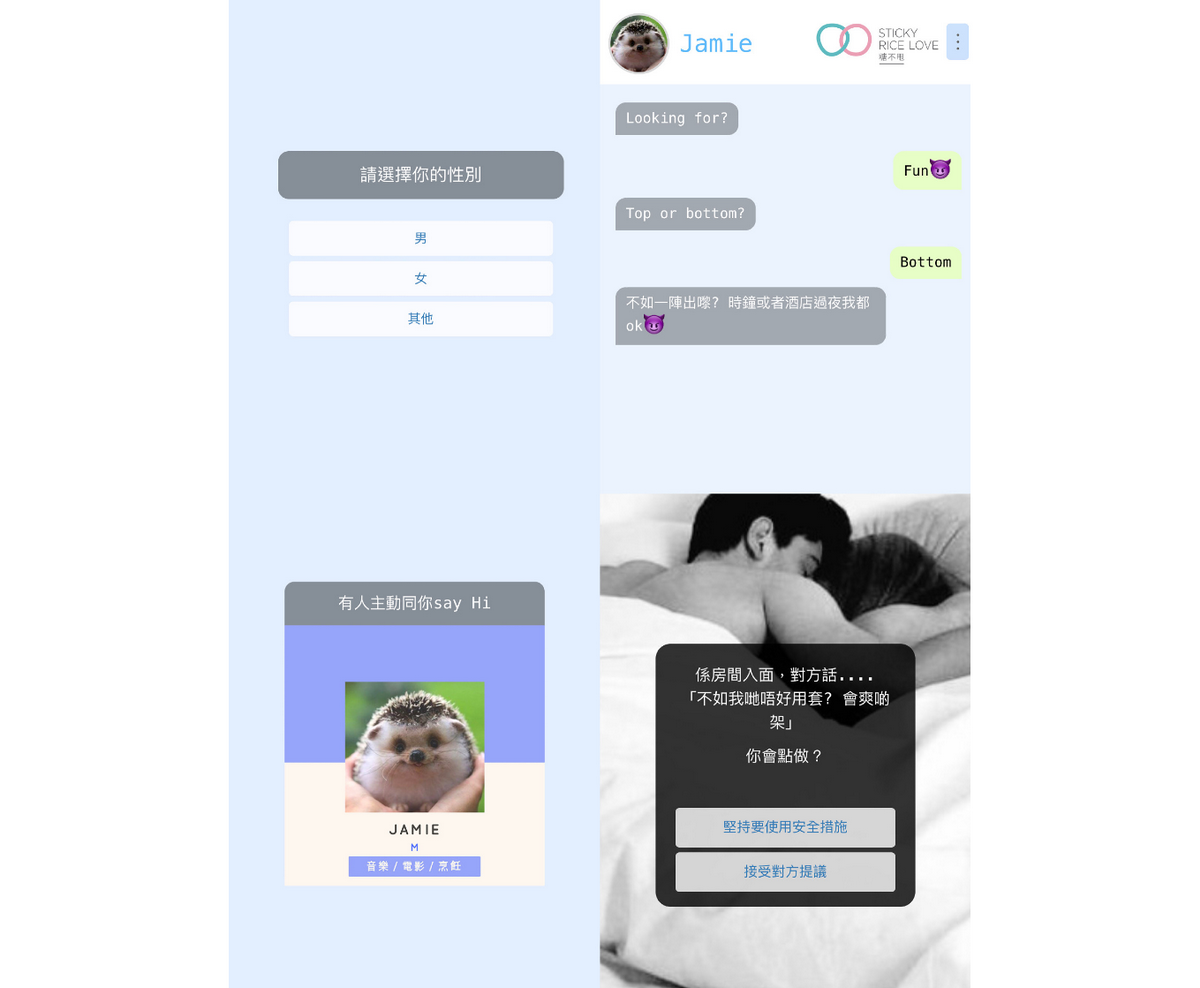
Screenshots of the simulation game.

**Figure 2:**
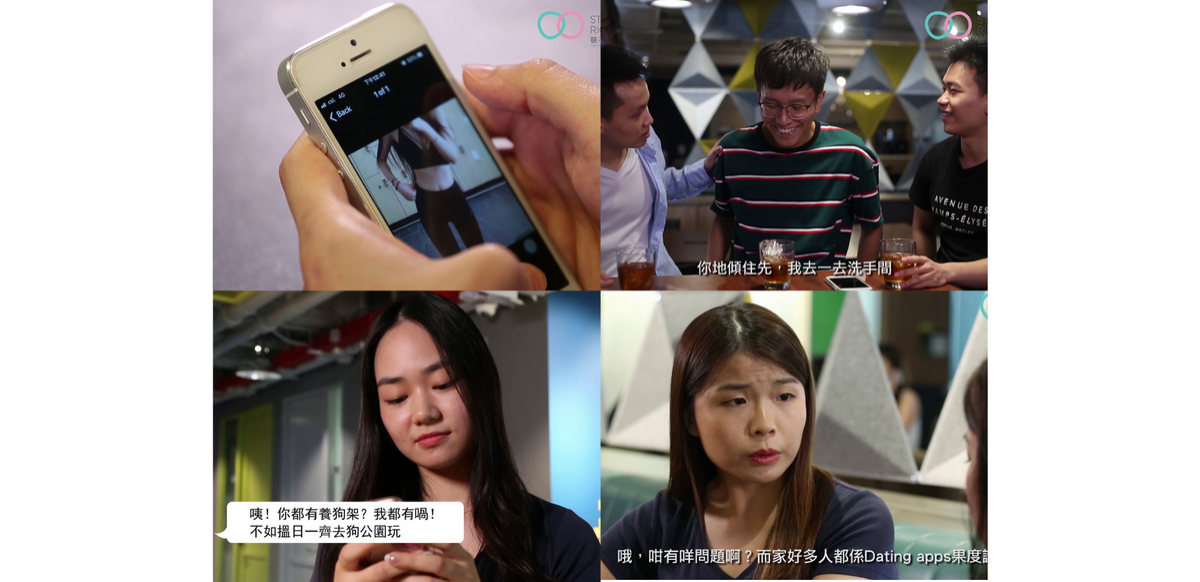
Screenshots of the short videos.

The final intervention programs were hosted online in the form of a website and are easily accessible by computer, smartphone, and tablet. Screenshots of the intervention programs can be found in Figures 1 and 2.

## Results

### The Intervention

The final intervention was comprised of four (2-4 minutes) short videos with points of discussion, an interactive scenario game (20-30 minutes), and a risk assessment tool. The videos aim to address the risks and benefits of using dating apps and encourage viewers to reflect on their perception of the dating apps used in the points of discussion. The first video illustrates similarities between meeting people on dating apps and in real life, providing examples of being misled by profile characteristics and monetary scams, the second video explains privacy concerns, the third demonstrates risky sexual behaviors associated with dating apps, and the fourth explains the legal issues and risks of sexual assault, including precautionary steps and available resources.

The scenario game is a first-person simulation game where the players are presented with multiple choices when faced with real-life scenarios. Players have to create a profile with attributes such as sexual orientation, gender, and types of relationship. Their choices generate a dating app profile, and chat bubbles will appear that contain messages from a virtual partner. The game is designed with various algorithms that result in both positive and adverse outcomes. The final page displays a brief summary explaining the culmination of the choices made.

The risk assessment tool consists of 14 questions that gives the participant a score to determine their risk level for adverse events when using dating apps. It covers five domains: sexual harassment, privacy, monetary and legal issues, and mental wellbeing.

### Pilot Study and Evaluation

Before full implementation, the interventional materials and processes were tested in a pilot study. Recruitment was done through university internal mass mail services for students, and criteria included prior experience with dating app use. We recruited 18 local college students aged between 18-25 years old, and then divided the participants into two small groups. We showed them the videos and invited them to play the game. They were then asked to fill out a postintervention questionnaire for general comments about the programs, which took about an hour altogether. We also held semistructured focus group discussions among the participants to collect further qualitative feedback about the intervention programs.

The feedback collected from the pilot was generally positive. Participants found the content of the intervention programs to be relatable and realistic for dating app users in Hong Kong, and also informative about the potential risks of dating app usage. Participants also offered useful suggestions about places for improvement, including enlarging the subtitles in the videos for a clearer read and modifying details in the app to make it more realistic. The intervention programs were revised based on these comments.

The effectiveness of the intervention will be further evaluated through a randomized control trial. The intervention will be disseminated among college students aged 18-25 years old in Hong Kong through engagements with public health officials, higher education institutions, and social media companies. The intervention delivery will be a combination of classroom and social media delivery.

## Discussion

### Principal Findings

Crowdsourcing was implemented as a core methodology and integrated into different parts of the development of the intervention. In the planning stage, crowdsourcing proved to be a useful method to get community input for public health issues. This is particularly important for emerging issues that have not been thoroughly researched in the existing literature. In the preparation of intervention materials, combining crowdsourcing with the peer-led approach also contributed significantly to designing programs that were more relatable and thus were more effective on the target population. Furthermore, crowdsourcing can also be a cost-effective tool since volunteers can do a considerable portion of the work.

Two key issues in adopting the crowdsourcing and peer-led approach are motivation and the extent of participation [12,13]. Brabham finds that a collection of motivators exists for crowdsourcing, including a love of community, the opportunity to develop one’s skills, and building a portfolio for future employment [13]. Apart from these factors, we observed that interest in the topic and relatability to personal experiences also played important roles in motivating the crowd to contribute to intervention development. We found that crowdsourcing is effective in empowering community members to take a more active part in developing the intervention, thus avoiding situations of nonparticipation like tokenism, decoration, and manipulation. The level of participation varies in different stages of the intervention development according to the aims of that particular stage. For stages involving brainstorming, assessment of needs and tastes of the target population, and design of creative content, we encouraged a high level of participation so the projects were peer-initiated, directed, and decided. For other stages that required professional knowledge, we also drew heavily on peer-generated data and ideas. This method has helped us to use crowdsourcing effectively to develop a peer-led intervention.

### Potential Challenges

During the intervention development process, the research team identified several challenges in applying crowdsourcing and the peer-led approach. One major challenge of the crowdsourcing approach is data management and analysis. Crowdsourcing could generate a considerable amount of loosely structured data, especially in the earlier stage of intervention development. This requires the research team to adopt effective strategies in data collection and interpretation. When eliciting ideas from the crowd, the research team should communicate the nature and objectives of the study, as well as the requirements for the content, to facilitate more relevant responses. To better interpret data, the research team also needs to develop a systematic analytical strategy that combines relevant theories with the existing literature, especially since the ultimate drafting of intervention outcomes and objectives will be the responsibility of the research team. One way to meet the challenge of data management is to combine large-scale, extensive crowdsourcing with small focus group discussions. This method can help ensure both the scope and depth of the data collected. The two sets of data can also complement each other and be compared for better accuracy.

Furthermore, while the PVCP approach was efficient in intensive face-to-face workshops, we found it challenging to sustain peer mentors’ interest and participation in the production over time. The production of creative content usually involves various stages and therefore lasts for weeks or even months. Because our peer mentors were all volunteers who had full-time occupations, it was impractical to ask for their full participation throughout the production process. As a result, parts of the production, especially the scriptwriting, were undertaken by the research team. Better engagement of peer mentors is needed in this step. Methods may include providing incentives or scheduling several workshops in key stages of the production.

Finally, and more generally, developing an intervention for dating app use in Hong Kong poses challenges in balancing between providing more information and perpetuating social stigma around dating app users. Due to conservative attitudes toward sex in the local context, dating app users have been stigmatized as casual, promiscuous, and immoral. Consequently, there is a fine line between raising awareness about risks in dating app use and further stigmatizing dating app users as immoral and irresponsible. To avoid the latter outcome, we adopted various methods, such as engaging dating app users in setting the intervention goals and addressing both benefits and risks in the intervention programs. However, it remains unclear whether these methods will be sufficient. Intervention developers should always consider cultural specificities and make their best efforts to eliminate any possible negative impacts brought by the intervention.

### Limitations

Several limitations should be considered in this study. First, the recruiting and promotion processes during the needs assessment, crowdsourcing contest, and peer-led intervention development were done through the research team’s network of nongovernmental organizations and a large general education course at a local university. While this allows the research team to target select groups of people who may have an interest in this topic, it may overlook other individuals who belong to other organizations or universities. Second, since the intervention development is heavily based on local contexts in Hong Kong, its results may not be generalizable in other places.

### Conclusion

This paper describes the evidence-based development of an intervention for safer dating app use using crowdsourcing and the peer-led approach. Our findings suggest that the advantages of crowdsourcing lay in its cost-effectiveness, its ability to collect extensive data, and its engagement of community members. We recommend that crowdsourcing be strategically used in various stages of intervention development.

This paper has also identified potential challenges in adopting the crowdsourcing approach, including data management and analysis and peer mentors’ participation in intervention material production. We suggest that intervention developers should pay special attention to the communication of program objectives during crowdsourcing and should work out a well-structured schedule to better sustain peer mentors’ interests throughout material production. Moreover, we have highlighted local cultural specificities as an important issue that should be taken into consideration during intervention development. The crowdsourced, peer-led model of intervention development harnesses both new technologies and collective intelligence. Therefore, we believe that it is particularly suitable to tackling new public health issues that have not been thoroughly researched in the existing literature.

## Appendix

Multimedia Appendix 1 Semistructured interview schedule (bilingual).

Multimedia Appendix 2 Themes from focus group discussions.

## Abbreviations

PVCP: Peer-Vetted Creative Production
