# Supplementary material for: Using Crowdsourcing to Develop a Peer-Led Intervention for Safer Dating App Use: Pilot Study
Source: JMIR Form Res. 2020 Apr 21;4(4):e12098. doi: 10.2196/12098 (PMC7201323; doi:10.2196/12098)
Supplement: Multimedia Appendix 1 [file formative_v4i4e12098_app1.docx]

# Appendix I Semi-structured interview schedule (bi-lingual)

1. Would you please describe your experience on using online dating?
   1. When did you/ your friend first start using dating apps?
      你幾時開始用
   2. dating apps?
   3. What dating app/s have you used? Who recommends it? How do you find out new online dating Apps?
      你用過邊啲dating apps? 有冇人推薦你用? 你係點樣發現新既dating apps?
   4. How was your experience when you first used a dating app?
      可唔可以分享一下你第一次用dating app嘅情況?
   5. How did you feel then?
      你覺得點?
   6. Was the experience same as what you had imagined?
      情況同你想像中嘅相唔相似?
2. How do people flirt and meet different onliners connected through the dating apps?
   1. Usually, who would initiate the conversations? You or the others?
      通常你會等其他人同你對話定你會主動搵人傾計?
   2. What would you consider before you start talking with them?
      係開始對話前你會考慮啲咩因素
   3. How do you know you have met someone you like through the dating app?
      你有冇係dating apps入面識到合適嘅人?
   4. What makes him/her attractive to you (What flips the coin)?
      你覺得對方有咩咁吸引? 點解你覺得對方係合適?
   5. What were you just looking for (sex)?  Agree what did learn about the on liners you were keen on.  Who did u decide which once you wanted to hook up with?
      你用dating apps係咪多數因為有性需要? 你會點去決定想約邊個出嚟?

      [if answered: no] Then why did you use dating apps?
      [如回答：不是]咁你用dating apps係因為咩原因？
   6. What makes you feel interested in the person on the other end of the phone?
      有啲咩會令到你對個個人有興趣?
   7. What makes you take this further?
      因為咩原因令到你想同佢再進一步發展?
   8. What do you do when you face conversations that make you feel uncomfortable (e.g. sexually explicit content)?
      有冇遇過一啲令你覺得尷尬／唔舒服嘅對話內容? 當時你點應對?
   9. How do you work out that the conversation and the person is genuine?
      你點判斷對方係講緊真話?
   10. At what stage you decide to meet in person?
       去到幾時／係咩情況下你覺得想／係時候同對方見面?
   11. Were you concerned about the potential dangers?
       你當時驚唔驚會有危險／中伏?
   12. What strategies you’d use to protect yourself? What kind of preparation did you do?
       你有冇做啲咩準備去保護自己?
   13. How did you feel when you two first met up?
       同對方見面嘅時候你覺得點?
   14. Was he/she the same as you imagined / expected?
       同你想像中嘅一唔一樣?
       [if answered： differently] How do you feel? How was the relationship later-on?
       [如回答：不一樣]當時你有咩感覺? 之後你哋嘅關係發展成點?
   15. Where there any surprises when you two met? How did you feel or react?
       當你哋見到面個陣有冇啲咩係你意料之外? 當時你有咩感受／反應?
3. Questions on online relationships, sex and love, highlighting sexual risks and harm reduction.
   1. How would you regard that relationship? Good or bad?
      你覺得你同佢[係dating apps上認識而有出來見面嘅人]嘅關係如何？
   2. What makes that relationship good or bad? What lessons have you learned?
      你覺得點先算係一段好／唔好嘅關係? 你從中有冇學到D咩?
   3. Can you describe an example of online relationship that you or your friend experiences and that worked well for you?
      你或者你身邊有冇人係係dating apps到識到另一半之後有好嘅結果?
   4. Can you describe a scary dangerous/ near-miss experience that you’d do differently when you look back?
      有冇發生過一啲恐怖／有驚無險嘅經歷? 如果比多次機會你，你會唔會做唔同嘅嘢?
   5. If sex took place, how did it happen? Were you satisfied with it?
      你哋有冇發生過任何親密行為? 係點發生?你滿唔滿意?
   6. Did sex happened under consent and was safe sex practiced?  And if not, why not?
      發生親密行為嘅時候係唔係雙方都自願? 你哋有冇用任何安全措施? 如果無，點解?
   7. Overall, how did you think about the experience of using dating app?
      你覺得用依啲apps識人嘅經歷係點嘅?
   8. What health information would you be interested to see in an app …in what from
      假設有個app係會提供一啲同建立關係／性健康有關嘅資訊, 你會唔會有興趣睇? 你覺得咩類型嘅資料／表達形式會吸引到你?
   9. What else would be useful on an app that you would use?
      你覺得app內有邊啲咩功能係特別有用?或者係一啲你覺得會有用而有需要增加嘅功能？
   10. Any other things about dating app that you would like to share? (for example, friends’ experience)
       有冇其他有關dating apps嘅嘢想分享? 可以係你朋友嘅經歷架。
