# Supplementary material for: Using Crowdsourcing to Develop a Peer-Led Intervention for Safer Dating App Use: Pilot Study
Source: JMIR Form Res. 2020 Apr 21;4(4):e12098. doi: 10.2196/12098 (PMC7201323; doi:10.2196/12098)
Supplement: Multimedia Appendix 2 [file formative_v4i4e12098_app2.docx]

Appendix II Themes from Focus Group Discussions

| **Theme** | **Sub-Themes** |  |
| --- | --- | --- |
| 1. **User Risk Taking Behaviours** | Legal risks | Underage sex |
|  |  | Drug taking |
|  |  | Sex in public places |
|  | Physical risks | Sexual health risks |
|  |  | Expediated encounters |
|  |  | Inconsistent condom use |
|  | Other risks: | Extortion |
|  |  | Exposure |
|  |  | Scams |
|  |  |  |
| 1. **Stigma & stereotyping** | Psychological distress | Perceived promiscuity of dating app users and unacceptance from friends |
|  | Barriers in seeking help | Lack of accountability with the police due to stigma and judgements |
|  |  | Gap in perspective between dating app users and advisory authorities |
|  |  |  |
| 1. **User Coping Strategies** | Safe sex practices | Strategic choice of meeting places |
|  | Personal Safety considerations | Trusting one’s instinct |
|  | Data and communications management/ blocking |  |
